# An Evolutionary Federated Learning Approach to Diagnose Alzheimer’s Disease Under Uncertainty

**DOI:** 10.3390/diagnostics15010080

**Published:** 2025-01-01

**Authors:** Nanziba Basnin, Tanjim Mahmud, Raihan Ul Islam, Karl Andersson

**Affiliations:** 1 Cybersecurity Laboratory, Luleå University of Technology, 97187 Luleå, Sweden; 2 Department of Computer Science and Engineering, Rangamati Science and Technology University, Rangamati 4500, Bangladesh; tanjim_cse@yahoo.com; 3 Department of Computer Science and Engineering, East West University, Dhaka 1212, Bangladesh; raihan.islam@ewubd.edu

**Keywords:** Alzheimer’s disease, convolutional neural network (CNN), federated learning, belief rule base, FedAvg, FedProx, genetic algorithm

## Abstract

**Background:** Alzheimer’s disease (AD) leads to severe cognitive impairment and functional decline in patients, and its exact cause remains unknown. Early diagnosis of AD is imperative to enable timely interventions that can slow the progression of the disease. This research tackles the complexity and uncertainty of AD by employing a multimodal approach that integrates medical imaging and demographic data. **Methods:** To scale this system to larger environments, such as hospital settings, and to ensure the sustainability, security, and privacy of sensitive data, this research employs both deep learning and federated learning frameworks. MRI images are pre-processed and fed into a convolutional neural network (CNN), which generates a prediction file. This prediction file is then combined with demographic data and distributed among clients for local training. Training is conducted both locally and globally using a belief rule base (BRB), which effectively integrates various data sources into a comprehensive diagnostic model. **Results:** The aggregated data values from local training are collected on a central server. Various aggregation methods are evaluated to assess the performance of the federated learning model, with results indicating that FedAvg outperforms other methods, achieving a global accuracy of 99.9%. **Conclusions:** The BRB effectively manages the uncertainty associated with AD data, providing a robust framework for integrating and analyzing diverse information. This research not only advances AD diagnostics by integrating multimodal data but also underscores the potential of federated learning for scalable, privacy-preserving healthcare solutions.

## 1. Introduction

In this age, 50 million people are globally suffering because of the impact of Alzheimer’s disease, and by 2050, it is predicted that this number will increase three-fold [1]. It is difficult to understand the actual causes of Alzheimer’s; however, studies show that genetics, environment, and lifestyle can have a profound impact [2]. In addition, this disease directly impacts cognitive functions and gradually impairs the ability of the patient to perform daily activities. With time, the patient may become prone to memory loss and abnormalities in speech and encounter confusion with the time and place they are in. Early-onset signs and symptoms of this disease can begin before the age of 65, while late-onset typically begins after 65 [3]. As a result, it is necessary to diagnose Alzheimer’s early so that the quality of life can be improved, adequate treatment can be acquired, and the progression of the disease can be slowed down, allowing the patient to seek psychological support to cope with the emotional impact of the disease. To achieve this purpose, an Alzheimer’s diagnosis system needs to be developed. Since the actual cause of Alzheimer’s is not clear and remains uncertain, the system should be able to deal with information not only limited to MRI scans but also about the person’s age, lifestyle, and environment. This will, in turn, tackle the complexity of the disease. In this research, a multidisciplinary approach where machine learning, the  belief rule base system [4], and federated learning [5,6] are employed. The machine learning model, namely the convolutional neural network, is customized so that it can achieve optimal accuracy when training the MRI image dataset of patients [7]. Furthermore, to overcome the uncertainty in identifying the exact stage of the disease, a trained belief rule-based model is developed by using evidential reasoning and particle swarm optimization [8]. Particle swarm optimization is used because it excels in finding optimal or near-optimal accuracy in complex, multi-dimensional search spaces. However, focusing solely on these models will not suffice for large-scale early diagnosis of Alzheimer’s. Implementing such a system in hospitals requires a broader approach, particularly addressing data sensitivity issues. Many hospitals are reluctant to share data due to privacy concerns. This is where federated learning—a form of decentralized system—becomes crucial. Federated learning enables the development of a large-scale diagnostic system without compromising data privacy, as it allows hospitals to collaboratively train models without sharing raw data [9]. This approach not only values the issue of patient data sensitivity but also leverages diverse datasets to improve diagnostic accuracy, making it a viable solution for widespread implementation in healthcare facilities.

This research intends to develop an Alzheimer’s disease diagnostic system wherein deep learning (convolutional neural network), knowledge representation (belief rule base system) [10], and federated learning technologies will be integrated. This is done to address the challenges with data uncertainty, diagnostic accuracy, privacy preservation of patient data, and reduced resource utilization.

### 1.1. Research Objectives

To address the research aims, the following research objectives have been formulated:

1. Systematically clean and curate datasets, including medical images and demographic data, for accurate Alzheimer’s disease (AD) diagnosis.
2. Design a convolutional neural network (CNN) architecture tailored for processing and classifying Alzheimer’s medical imaging data.
3. Combine image data with demographic information to improve diagnostic accuracy.
4. Develop an Optimized trained belief rule base (BRB) to handle data uncertainties.
5. Incorporate the belief rule base (BRB) system in the federated learning framework.
6. Compare and evaluate the different (i.e., FedAvg, FedProx, and genetic algorithm) aggregators used in the federated learning model.

### 1.2. Research Questions

1. How to develop a pre-processing pipeline combining medical images and demographic data for enhanced Alzheimer’s disease diagnosis?
2. How to design a CNN architecture optimized for Alzheimer’s medical imaging data classification?
3. How to integrate image and demographic data to improve diagnostic accuracy?
4. How to establish a federated learning framework with a belief rule base to handle data uncertainties?
5. How does advanced federated learning for medical applications improve privacy-preserving diagnostic frameworks?
6. How can multimodal data handling and uncertainty management in machine learning for medical diagnostics improve accuracy and overcome uncertainty?

## 2. Related Work

Machine learning is transforming the healthcare system, offering new advancements and solutions. Alzheimer’s disease (AD), affecting millions worldwide, remains a critical area where these technologies are making a significant impact. It is essential to detect AD in its stages to slow down its progression through proper care and treatment. Consequently, there has been a rise in the use of artificial intelligence (AI) techniques [1,3] due to their capacity to analyze datasets and recognize complex patterns. This section will delve into research on AI-driven Alzheimer’s diagnosis focusing particularly on learning methods such as convolutional neural networks (CNNs) and the application of federated learning in this field. Several studies have provided insights into classifying Alzheimer’s disease using data types machine learning approaches and federated learning techniques [5].

### 2.1. Machine Learning to Classify AD Using MRI Images

The XAI framework in this study uses the mapping of occlusion sensitivity, emphasizing white matter hyperintensities (WMHs) in the image data [11]. The deep learning classifier, namely EfficientNet-B0, achieves an accuracy of 80.0%. This work [12] integrates image data with the XAI frameworks of Saliency Map and Layer-wise Relevance Propagation, analyzing MRI, 3D PET, biological markers, and assessments. This study will make use of a DL 3D CNN AD classifier. This research [12] incorporates image data and XAI frameworks such as Saliency Map and Layer-wise Relevance Propagation (LRP), analyzing MRI, 3D PET, biological markers, and assessments. A DL 3D CNN AD classifier is employed for this study. This study [13] involves image data and XAI frameworks utilizing decision trees (DTs). Significant features include demographic data, cognitive factors, and brain metabolism data. Classifiers include Bernoulli naive Bayes (NB), SVM, kNN, random forest (RF), AdaBoost, and gradient boosting (GBoost), achieving an accuracy of 91.0%. Significant features, including demographic data, cognitive factors, and brain metabolism data, are highlighted in this study [13], which utilizes decision trees (DTs) as the XAI framework. The classifiers, Bernoulli naive Bayes (NB), SVM, kNN, random forest (RF), AdaBoost, and gradient boosting (GBoost) collectively achieve an accuracy of 91.0%. In this research, demographic data, cognitive factors, and MRI image data are combined [13]. The data are passed through a decision tree (DT) as the XAI framework. Other classifiers, such as Bernoulli naive Bayes (NB), SVM, kNN, random forest (RF), AdaBoost, and gradient boosting (GBoost) collectively achieve an accuracy of 91.0%. In [14], an XAI framework was employed wherein a 3D ultrametric contour map is deployed alongside a 3D class activation map and 3D GRadCAM, all to analyze the image data. As we understand, there are significant features which can be accurately classified after using the 3D CNN deep learning model. Overall, and accuracy of 76.6% was achieved. This work employed sensitivity analysis and occlusion as XAI frameworks in the analysis of 3D image data. The accuracy of the DL 3D CNN classifier is 77.0% [15]. The paper by [16] classifies healthy controls versus Alzheimer’s disease subjects based on numeric data. LIME and SHAP are used as XAI frameworks, while some of the features identified include whole brain volume, years of education, and socio-economic status. Classifiers used include support vector machine (SVM), k-nearest neighbors (kNN), and multilayer perceptron. For the classification of HC versus AD, this study [17] leverages XAI frameworks such as HAM and PCR, focusing on salient AD-related features like cerebral cortex and hippocampus atrophy. The DL CNN classifier achieves an impressive accuracy of 95.4%. In [17], healthy controls (HCs) were compared to Alzheimer’s disease (AD) classification using XAI frameworks (i.e., HAM and PCR). These frameworks focus on features such as the cerebral cortex and hippocampus atrophy through the deep learning (DL) convolutional neural network (CNN), achieving an impressive accuracy of 95.4%. This study [18] focuses on classifying healthy controls (HCs), individuals with mild cognitive impairment (MCI), and Alzheimer’s disease (AD) using brain image data. It employs the XAI framework GNNExplainer to interpret predictions made by a deep learning graph neural network (GNN). Key features include brain region volume, cortical surface area, and cortical thickness. The GNN classifier achieves an accuracy of 53.5 ± 4.5%, providing insights into the important brain features associated with these conditions. Marwa et al. [19] utilized a deep neural network (DNN) pipeline on a dataset of 6400 MRI images to effectively identify various stages of Alzheimer’s disease, achieving a remarkable accuracy of 99.68%. Ghazal et al. [20] developed a classification model using transfer learning to categorize Alzheimer’s disease into Mild Demented, Moderate Demented, Non-Demented, and Very Mild Demented stages, employing AlexNet and obtaining a simulation accuracy of 91.7%. AlSaeed et al. [21] proposed a CNN-based model, ResNet50, for automatic feature extraction from MRI images to detect Alzheimer’s disease. Their model, tested against Softmax, SVM, and RF models, showed superior performance with an accuracy ranging from 85.7% to 99% on the MRI ADNI dataset. Hamdi et al. [22] introduced a CAD system based on a CNN model for Alzheimer’s detection using the MRI ADNI dataset, achieving 96% accuracy. Helaly et al. [23] employed both CNN and VGG-19 transfer learning models to classify Alzheimer’s stages with the ADNI dataset, with VGG-19 achieving an accuracy of 97%. Mohammed et al. [24] utilized AlexNet, ResNet-50, and hybrid models combining AlexNet+SVM and ResNet-50+SVM to diagnose Alzheimer’s using the OASIS dataset, with AlexNet+SVM achieving the highest accuracy of 94.8%. Pradhan et al. [25] compared VGG-19 and DenseNet architectures for Alzheimer’s classification, finding VGG-19 to perform better. Salehi et al. [26] implemented a CNN model to diagnose Alzheimer’s early using the MRI ADNI dataset, achieving a 99% accuracy. Suganthe, Ravi Chandaran et al. [27] employed deep CNN and VGG-16-based CNN models for Alzheimer’s detection using the ADNI dataset, both showing excellent accuracy. Hussain et al. [28] proposed a 12-layer CNN model for Alzheimer’s classification using the OASIS dataset, achieving 97.75% accuracy, outperforming other existing CNN models on this dataset. Basaia et al. [29] applied a CNN model to the ADNI dataset for Alzheimer’s detection, achieving high performance with both ADNI and combined ADNI+non-ADNI datasets. Ji et al. [30] proposed an ensemble learning approach using deep learning for early Alzheimer’s diagnosis with the MRI ADNI dataset, achieving up to 97.65% accuracy for AD/mild cognitive impairment stages. Islam et al. [31] proposed a CNN model for Alzheimer’s detection and classification, evaluated on the OASIS dataset. Their model is noted for its speed, lack of reliance on handcrafted features, and suitability for small datasets.

Table 1 briefly highlights the use of various machine learning models in the classification of Alzheimer’s disease. Overall, a deduction is made that convolutional models used by Yu et al. [17] and De et al. [12] can achieve a high accuracy when it comes to image classification tasks that involve MRI scans. Yu et al. further revealed an impressive accuracy of 95.4% in differentiating between a healthy control group and Alzheimer’s disease. Complex features, such as those found in MRI scans highlighting brain atrophy, are effectively extracted and learned by CNNs, making them ideal for medical imaging tasks. In contrast, traditional models, such as decision trees and support vector machines, are limited in handling complex tasks. Unlike CNN, the problem with a traditional model is that it requires feature engineering which can be computationally expensive, and training large amounts of data can be slow. Furthermore, CNN is open to adding features of explainability such as the use of GradCAM and Layer-wise Relevance Propagation (LRP) which can enhance the CNN’s ability in the process of decision-making by adding more transparency. Explainable AI can be a future direction for this current research. In conclusion, CNNs, according to the reviewed literature, have consistently outperformed traditional machine learning models when it comes to accuracy and scalability, particularly when applied to large and complex datasets.

### 2.2. Federated Learning-Based Solution for Building Alzheimer’s Diagnosis System

Khalil et al. [5] built a hardware acceleration approach to expedite the training and testing of their FL model. In this approach, the hardware accelerator is constructed using VHDL hardware description language and an Altera 10 GX FPGA. Simulation results indicate that the proposed methods achieved accuracies of 89% and sensitivities of 87% for early detection, outperforming other state-of-the-art algorithms in terms of training time. Moreover, the proposed algorithms exhibited a power consumption range of 35–39 mW, rendering them suitable for resource-constrained devices (see Table 2).

Mitrovska et al. [6] employed two algorithms, namely Federated Averaging (FedAvg) and Secure Aggregation (SecAgg) which were combined, and their performance was compared with a centralized ML model training. By simulating such diverse environments, the influence of demographic factors (such as sex, age, and diagnosis) and imbalanced data distributions was investigated. These simulations made it possible to assess the effect of statistical variations on ML models trained with FL, underscoring the significance of accounting for these differences in training models for AD detection. From the end result, it was demonstrated that, when FL is combined with SecAgg, it ensures privacy, as evidenced by simulated membership inference attacks showcasing its privacy guarantees.

Trivedi et al. [32] developed a streamlined federated deep learning framework for classifying Alzheimer’s diseases, ensuring data privacy using IID datasets in a client-server setup. Evaluations with single and multiple clients under traditional and federated learning scenarios revealed AlexNet as the best pre-trained model, achieving 98.53% accuracy. The framework was then tested with IID datasets using federated learning (FL).

Altalbe et al. [33] developed a deep neural network (DNN) within a federated learning framework to detect and diagnose brain disorders. The dataset used here is from the Kay Elemetrics voice disorder database, which was divided among two clients, to built separate training models. Overfitting was reduced through three review rounds by each client. The model identified brain disorders with an accuracy of 82.82%, preserving privacy and security.

Mandawkar et al. [34] engineered a federated learning-based Tawny Flamingo deep CNN classifier to classify Alzheimer’s disease. The classifier processes clinical data from multiple distributed sources and delivers high accuracy. Tuned using the Tawny Flamingo algorithm, the model achieved accuracies of 98.252% in K-fold analysis and 97.995% in training percentage-dependent analysis for detecting Alzheimer’s disease.

Castro et al. [35] deployed an Alzheimer’s diagnosis system by using MRI brain images wherein federated learning (FL) was integrated for biometric recognition validating image authentication. This approach aimed to protect privacy and prevent data poisoning attacks. Experiments conducted on the OASIS and ADNI datasets demonstrated that the system’s performance was comparable to that of a centralized ML system without privacy measures.

Qian et al. [9] introduced FeDeFo, which stands for a federal deep forest model utilized for calculating hippocampal volume using sMRI images to classify Alzheimer’s disease. This uses a federated learning framework to train a gradient-boosting decision tree (GBDT) model leveraging local client data to ensure data privacy. Furthermore, this approach addresses data discrepancies, by incorporating a deep forest model, combined with the federated GBDT to personalize the model for each client. Experiments demonstrated that this method effectively personalized the model while protecting data privacy, providing a new method for Alzheimer’s disease classification. Table 1 presents a key summary of studies in Alzheimer’s disease detection using FL.

### 2.3. Addressing the Existing Research Gaps

In summary, future studies should prioritize developing FL models which are capable of integrating data from multiple sources, as this will make the model more generalized. It is also necessary to explore the use of evolutionary algorithms in FL. These algorithms will not only help to optimize model parameters but also improve the performance in areas wherein data are complex and environments are distributed. As a result, methodologies incorporating these evolutionary algorithms should be built to handle data heterogeneity and uncertainty. This will ensure that FL frameworks maintain optimal accuracy and fairness across different client datasets. In scenarios wherein data are distributed across multiple servers, applying advanced privacy-preserving techniques, such as differential privacy and multi-party computation, it is crucial to ensure the security of sensitive patient data in an FL system. In addition, FL frameworks should showcase scale-able behavior to manage large-scale deployment. Furthermore, FL systems should be deployed in a clinical setting to carry out clinical trials in the real world to test their validity and applicability. In order to provide more evidence for effectiveness in real-world healthcare settings, there is also a requirement to perform more research on the aspect of expanding hardware acceleration in FL.

Hence, this research will attempt to address most of the research gaps by introducing evolutionary algorithms and optimization techniques to enhance the performance of FL framework.

## 3. Methods

**Figure 1:**
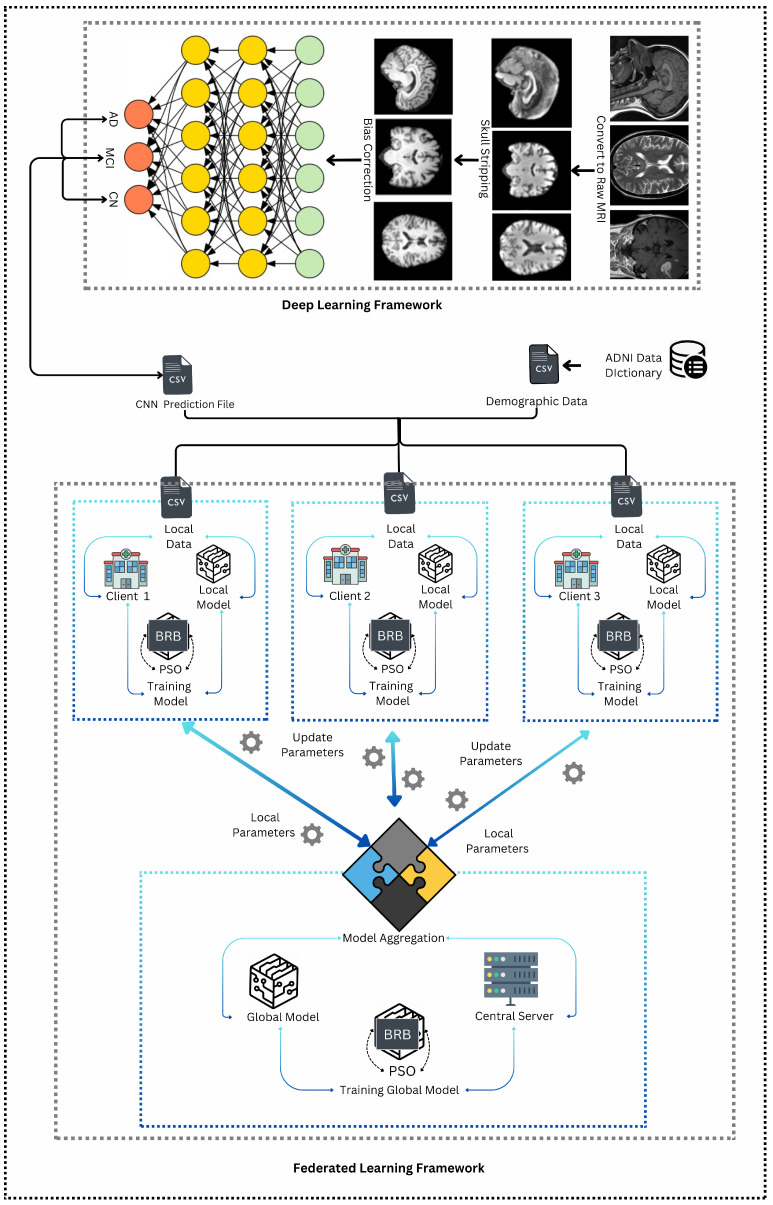
Operational framework.

Figure 1 illustrates the operational framework used to develop the system in this research. From the figure, it can be deduced that this framework is a hybrid system, as it combines two distinct methodologies: deep learning framework and federated learning framework [5,6]. In the first methodology, MRI images undergo pre-processing steps such as skull stripping and bias correction to improve image quality. After pre-processing the images, the dataset is passed as an input to the convolutional neural network (CNN), for the purpose of classification of Alzheimer’s disease (AD). Thus, the CNN is trained to classify Alzheimer’s disease into three categories: Alzheimer’s Disease (AD), Mild Cognitive Impairment (MCI), and Cognitively Normal (CN). Afterwards, the results from the classification are converted into a CSV file that contains the predicted classes for each subject.

This CSV file is then merged with demographic data received from the ADNI dataset mentioned in Section 3.1. This results in a multi-modal dataset that integrates both clinical and demographic information. The combined dataset is subsequently split equally into three parts, each corresponding to a different client in the federated learning framework. In this second methodology, the three clients can be potentially considered as three hospitals where the local models are trained using a belief rule base (BRB) system [36]. The BRB system is enhanced with particle swarm optimization (PSO) to optimize the parameters [37]. Each client trains its BRB model locally, and the resulting parameters are then sent to a central server. At the server, these local parameters are aggregated to update the global model (which uses the same BRBs). This federated learning process is iterative, where the central server sends the updated global model parameters back to the clients. The clients then use these updated parameters to refine their local models in the next round. This iterative process continues—typically for three iterations—until the global model reaches an optimal state, effectively capturing the combined knowledge from all local models and providing a robust predictive tool for Alzheimer’s disease classification.

### 3.1. Description of Dataset Applied in This Research

For this research, data were obtained from the Alzheimer’s Disease Neurology Initiative (ADNI) [38]. The ADNI dataset includes a variety of modalities, making it an ideal resource for researchers aiming to utilize it for the early detection of Alzheimer’s disease. This dataset comprises both demographic information and imaging data of patients. The fMRI dataset consists of images provided in DICOM and NIFTI formats.

DICOM images resemble a series of frames, similar to those found in videos, whereas NIfTI images provide a comprehensive representation of a specific part of the human body; in this case, the brain. These images include all slices captured by scanners or other imaging methods. The NIfTI format is converted to enable access to raw MRI images.

The dataset contains a total of 5182 images, categorized into three classes: “Alzheimer’s Disease (AD)”, “Mild Cognitive Impairment (MCI)”, and “Cognitively Normal”. After pre-processing the data, the dataset was divided into training and testing sets with an 80:20 split ratio.

### 3.2. Steps of MRI Dataset Preprocessing

The conversion of NIfTI files into raw images involves multiple steps that differ from standard image formats such as PNG or JPG. For this process, FSL software (Version v6 2018) is utilized, installed via the Windows Subsystem for Linux (WSL) and added to the PATH environment on a Windows system. The pre-processing pipeline consists of affine registration, skull stripping, and bias correction, each executed sequentially to prepare the MRI data for further analysis.

Affine Registration:

Affine registration determines the optimal transformation that aligns points in one image with corresponding points in another. This transformation includes translation, scaling, rotation, and shearing, offering greater alignment flexibility compared to rigid or similar transformations. The process begins by selecting a reference NIfTI file from the FSL/data/standard directory. Depending on the specifications of the MRI files, the appropriate MNI152 file is chosen. For example, if the MRI files are 1 mm T1 MRIs, the MNI152_T1_1mm.nii file is selected. The reference file is then placed in the atlas folder, and the register.py file in the pre-processing folder is updated. Executing the register.py script in the terminal generates the ADNIReg folder, which contains subdirectories (AD, CN, MCI) with the transformed NIfTI files.

Skull Stripping:

MRI scans often contain extraneous details, such as the skull, eyes, nose, and other facial features, which must be removed to isolate the brain region. Skull stripping eliminates this unnecessary data, ensuring that they do not interfere with the machine learning model’s training or accuracy. The bet tool from the FSL library is used for this purpose. The resulting files are saved in a newly created ADNIBrain folder while preserving the subdirectory structure (AD, CN, MCI).

Bias Correction:

N4 bias correction is applied to address intensity inconsistencies, also known as bias fields, present in medical images. These inconsistencies, caused by scanner settings or physical structures of patients, can compromise the accuracy of image analysis. By eliminating bias fields, the clarity and reliability of the images are improved. The Advanced Normalization Tools (ANTs) library is employed for this step. Since this transformation is computationally intensive, the Python script can be modified to process images in batches by adjusting the batch size accordingly.

By implementing these pre-processing steps, the MRI data are effectively prepared for further analysis, ensuring precision and efficiency in subsequent processing stages.

### 3.3. Deep Learning Framework

After the MRI images undergo pre-processing, the dataset is randomly divided into 80% training and 20% testing, to integrate into the deep learning model, namely the convolutional neural network. The architecture is demonstrated as follows:

#### Modified Convolutional Neural Network

Based on the specified CNN model architecture for diagnosing Alzheimer’s disease from imaging data, multiple convolutional layers are incorporated to extract features from the input images. Deep learning techniques are leveraged to capture complex patterns within the data. The model starts with a sequence of convolutional layers, each followed by max pooling and batch normalization. The convolutional layers apply filters to detect features such as edges and textures in the images, with these features becoming increasingly abstract as they progress through the layers. Max pooling layers reduce the spatial dimensions of the data, which decreases the computational burden and mitigates the risk of overfitting. Batch normalization is used to stabilize the learning process by normalizing activations, making the network more robust to variations in the input data distribution. This structured combination of layers enables the model to effectively learn and generalize to new imaging data.

Dense layers with dropout are also included in the model to further refine the features extracted by the convolutional layers into meaningful patterns suitable for classification (See Table 3). Dropout randomly deactivates a fraction of the neurons during training, preventing overfitting by ensuring that the model does not become excessively reliant on any specific feature. The final output layer employs a softmax activation function to generate probabilities for each of the four classes, providing confidence scores for the potential diagnoses. The Adam optimizer and Sparse Categorical Cross-Entropy loss function are utilized to facilitate efficient and precise learning by adjusting the model weights to minimize the error between the predicted and actual class labels.

Overall, this CNN architecture delivers a robust and accurate approach for diagnosing Alzheimer’s disease from imaging data. By leveraging advanced deep learning techniques, the model achieves high performance and reliability.

### 3.4. Federated Learning Architecture

Federated learning enables the decentralized training of large-scale models without accessing user data, thus ensuring privacy. Federated learning is classified into Horizontal Federated Learning (HFL), Vertical Federated Learning (VFL), and Federated Transfer Learning (TFL). In this research, HFL is adopted, enabling multiple clients to jointly train a global model while ensuring the privacy of their local data. The steps involved in the HFL framework are described as follows:

#### 3.4.1. Data Preparation

**Figure 2:**
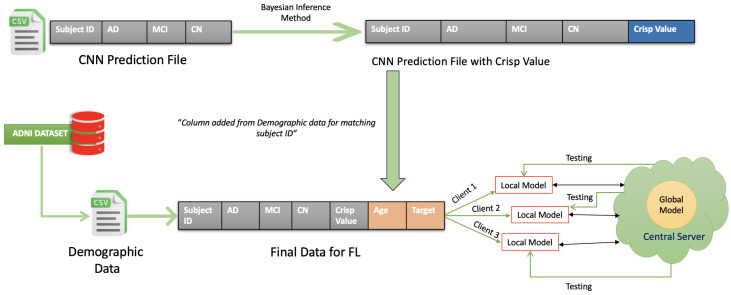
Data preparation.

Figure 2 illustrates how the data preparation process, essential for implementing a federated learning (FL) framework, is carried out for diagnosing Alzheimer’s disease. This process combines demographic information with CNN-generated predictions and distributes the resulting dataset across multiple clients while ensuring data privacy is maintained.

Step 1: Preparing and Matching Demographic Data

Initially, the demographic data such as patients’ ages and patient ID are extracted from the Alzheimer’s Disease Neuroimaging Initiative (ADNI) dataset. The duplicated values are excluded in the CSV file. The CNN predictions—providing the probabilities for Alzheimer’s stages such as Alzheimer’s Disease (AD), Mild Cognitive Impairment (MCI), and Cognitively Normal (CN)—are gathered into another CSV file. The demographic data are then matched to the CNN predictions by using the Subject ID.

Step 2: Calculating the Crisp Value Using the Utility Function

Once the data have been matched, a “Crisp Value” is calculated for each patient. This is performed by applying a utility function, which combines the predicted probabilities of the Alzheimer’s stages with their corresponding utility scores. The utility function is as follows:

ExpectedUtility=P(CN)·U(CN)+P(MCI)·U(MCI)+P(AD)·U(AD)

Here, P(CN), P(MCI), and P(AD) are the predicted probabilities for Cognitively Normal, Mild Cognitive Impairment, and Alzheimer’s Disease, respectively. Meanwhile, U(CN), U(MCI), and U(AD) represent the utility values for each stage. These utility values are derived from [39], with scores ranging from 0.2 for severe Alzheimer’s to 0.7 for mild cognitive impairment.

Step 3: Horizontal Partitioning of Data Across Clients

Once the Crisp Values are calculated and the data are enriched, the dataset is horizontally partitioned. This means that the data are split into subsets with identical columns but different rows. These subsets are distributed to various clients (Client 1, Client 2, Client 3), allowing them to train their models locally. By ensuring that raw data are never exchanged between clients or with a central server, this process effectively maintains patient privacy.

By integrating CNN predictions with demographic data and applying utility-based calculations, this system improves the accuracy and nuance of Alzheimer’s diagnosis. Horizontal partitioning ensures data privacy is preserved within a federated learning setup, making this approach both practical and secure for real-world healthcare applications.

#### 3.4.2. Local Training with BRB and PSO

Each client independently trains a local model using the belief rule base (BRB) mechanism combined with particle swarm optimization (PSO). The belief rule base (BRB) incorporates uncertainty by assigning belief degrees to different possible outcomes based on input variables such as CNN values and age. These belief degrees represent the level of confidence in each classification, allowing the model to account for uncertainty in the data. The rules in the BRB assess the likelihood of different conditions (such as CN, MCI, AD) and help manage any uncertainty arising from incomplete or noisy data. In the training process, each client in the federated learning setup independently trains a local model using the BRB mechanism. The belief rule base parameters are optimized using particle swarm optimization (PSO), which helps the model better handle data uncertainties and improve classification performance. After local training, the parameters are shared with a central server, where they are aggregated and refined to create a global model. This ensures that the model learns from data across different clients while maintaining privacy. This combination allows the model to handle uncertainties in the data effectively and optimize the parameters for better performance.

#### 3.4.3. Belief Rule Base (BRB) Mechanism

**Figure 3:**
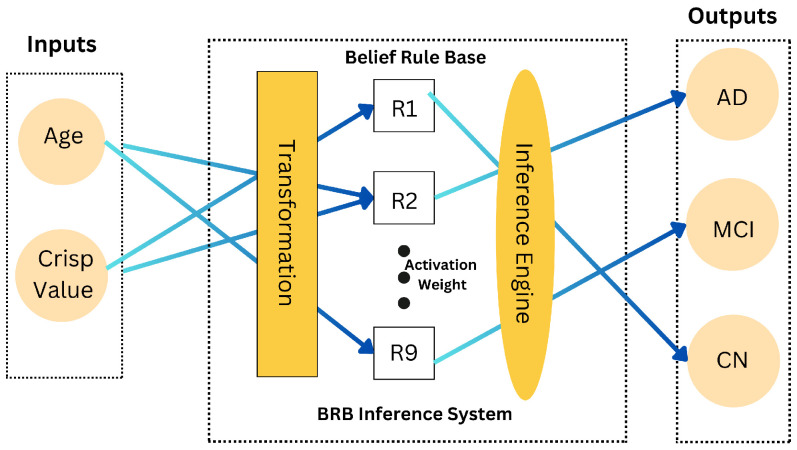
Belief rule base system architecture.

The BRB mechanism is central to our model. It involves defining rules that map input variables to belief degrees for classification outcomes (see Figure 3). Each rule $R_{i}$ is formulated to assess the probability of a patient’s condition based on their CNN value and age. The rules and their weights are applied to determine the likely classification outcome. Table 4 presents the initial rule base used in our framework, detailing the rules, their weights, and activation weights.

The rules in Table 4 serve as the foundation for the BRB framework, guiding the decision-making process by evaluating the likelihood of each classification outcome (CN, MCI, AD) based on the input variables (CNN_Value, Age).

The BRB mechanism is fundamental for managing uncertainties and variability in the data. It encompasses several key components [4,36]:

Rule Structure:

Each rule $R_{i}$ in the BRB maps input variables (e.g., CNN values, age) to belief degrees for possible outcomes (CN, MCI, AD):

(1) $$R_{i}:\mathrm{IF}\;\left(X_{1}\in A_{i}\right)\;\mathrm{AND}\;\left(X_{2}\in B_{i}\right)\;\mathrm{THEN}\;\left(\beta_{i1},\beta_{i2},\beta_{i3}\right)$$

Belief Degrees and Rule Weights:

Belief degrees $\beta_{ij}$ quantify the confidence in each classification outcome given a rule $R_{i}$. These degrees must sum to 1 for each rule:

(2) $$\sum_{j=1}^{3}\beta_{ij}=1$$

Rule weights $w_{i}$ indicate the importance of each rule $R_{i}$ and are normalized such that

(3) $$\sum_{i=1}^{N}w_{i}=1$$

where *N* is the total number of rules.

Attribute Weights:

Attribute weights $\lambda_{k}$ reflect the significance of each input variable $X_{k}$ in influencing the belief degrees. They adjust the impact of each variable on the matching degree calculation.

Analytical Process of BRB:

The analytical process of the belief rule base (BRB) involves the following steps: input transformation, calculation of the matching degree, belief update, and output aggregation.

#### 3.4.4. Local Parameter Optimization

The local model parameters are updated by minimizing the local objective function $L_{i}$ using PSO. The optimization process ensures that the model parameters converge to an optimal solution that reflects the local data characteristics [8,37]. The optimization process is defined as

(4) $$\theta_{i}^{t}=\mathrm{Optimize}\left(\theta_{i}^{t-1},D_{i}\right)$$

where $\theta_{i}^{t}$ represents the optimized parameters at iteration *t*, and $D_{i}$ is the dataset for client *i*.

#### 3.4.5. Parameter Sharing and Aggregation

After completing the local training, the clients transmit their updated BRB parameters to the central server. The gradients $\nabla \theta_{i}^{t}$ from each client are then aggregated to update the global model parameters $\theta_{G}^{t+1}$.

(5) $$\theta_{G}^{t+1}=\theta_{G}^{t}-\alpha_{\theta }\sum_{i=1}^{N}\nabla \theta_{i}^{t}$$

Here, $\alpha_{\theta }$ is the learning rate and *N* represents the number of clients. This aggregation process ensures that each client’s data influence the global model proportionally.

#### 3.4.6. Global Model Update and PSO Optimization

The central server retrains the global model using the aggregated BRB parameters and further optimizes them using PSO. This iterative refinement of parameters helps to achieve a consistent global model across all clients. The updated global parameters are then sent back to the clients:

(6) $$\left[h!\right]\theta_{i}^{t+1}\leftarrow \theta_{G}^{t+1}$$

#### 3.4.7. Iterative Optimization with PSO

**Figure 4:**
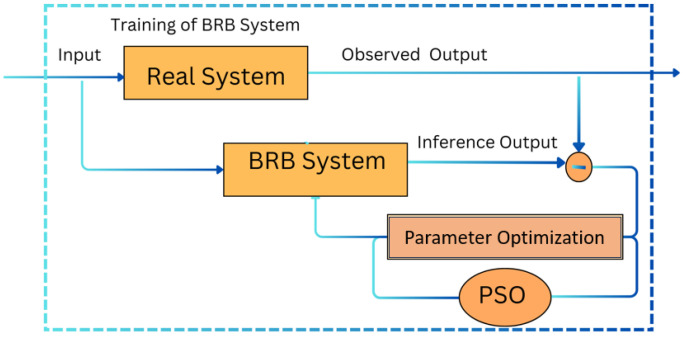
Trained belief rule base system.

This iterative process, which involves local training, parameter sharing, and global model updating, is repeated for five iterations to ensure faster convergence and efficient training (see Figure 4). The detailed steps of the PSO optimization process are outlined in Algorithm 1.
**Algorithm 1** Federated Learning with Particle Swarm Optimization (PSO) for Alzheimer’s Disease Diagnosis using FedAvg.1:**Input:**Dataset: ’CNN_Value’, ’Age’, ’Group’Clients: 3Iterations: *num_iterations*PSO params: *swarmsize*, *maxiter*2:**Output:** Optimized BRB parameters, classification results3:Load and split the dataset into train/test sets.4:Divide data into subsets for 3 clients.5:Scale features ’CNN_Value’ and ’Age’ using MinMaxScaler.6:Save processed data to CSV files.7:**BRB Model:** transform_inputvalue, high, low8:**return**$\left(\frac{\mathrm{high}-\mathrm{value}}{\mathrm{high}-\mathrm{low}},\frac{\mathrm{value}-\mathrm{low}}{\mathrm{high}-\mathrm{low}}\right)$calculate_degreevalue, ref_high, ref_low, beliefs9:(high,low)←transform_inputvalue, ref_high, ref_low10:**return**$\left(\mathrm{beliefs}{\left[0\right]}^{\mathrm{high}}\times \mathrm{beliefs}{\left[1\right]}^{\mathrm{low}}\right)$brb_modelparams, inputs11:Compute belief degrees and weights.12:Compute matching degrees and update beliefs.13:**return** aggregated output.evaluate_modelparams, data14:**return** Mean Squared Error of predictions.15:**Optimization:**16:**for**iteration←1 to *num_iterations* **do**17:   **for** each client **do**18:     Run PSO for the client and obtain parameters.19:     Ensure belief degrees sum to 1.20:     Evaluate train/test MSE and accuracy.21:     Save client-specific results.22:   **end for**23:   Average parameters across clients.24:   Refine parameters with PSO.25:   Evaluate on full dataset.26:   Save final results.27:**end for**28:**Output:** Final parameters and evaluation results.

This framework demonstrates the integration of federated learning, the BRB mechanism, and PSO for effective Alzheimer’s disease diagnosis. The iterative process of local training, parameter sharing, and global model updating ensures efficient training and accurate diagnostic results. The detailed structure and mechanisms of the BRB ensure that the system can handle data variability and uncertainties effectively. This Algorithm 1 integrates federated learning (FL) with particle swarm optimization (PSO) to optimize a belief rule base (BRB) model aimed at diagnosing Alzheimer’s disease. The key steps are as follows:

First, the dataset, consisting of features such as “CNN_Value”, “Age”, and “Group”, is divided among three clients. Each client scales the data using a MinMaxScaler, processes it, and stores the results for use in the federated learning process.

In the BRB model, functions are defined to transform input values into belief degrees and calculate matching degrees, which are used to compute the overall belief for a diagnosis. The model’s performance is assessed using Mean Squared Error (MSE) to ensure accuracy.

During the optimization phase, each client independently applies PSO to tune the BRB parameters. The belief degrees are normalized, and the model’s accuracy is evaluated on training and testing data. After each iteration, the parameters from all clients are averaged using the FedAvg technique to create a global model.

The global model undergoes further refinement with PSO, and its performance is evaluated on the entire dataset. Finally, the algorithm outputs the optimized parameters and classification results, which can be used to diagnose Alzheimer’s disease more effectively.

## 4. Results

### 4.1. Libraries and Packages for System Implementation

The Alzheimer’s disease prediction system was developed using a comprehensive set of Python libraries, each serving specific roles in the process from data preparation to model deployment. The system started by converting MRI images from the NIFTI format to raw PNG files using libraries like Nibabel, NumPy, and PIL, enabling the images to be used as input for the convolutional neural network (CNN). The CNN, built with TensorFlow and Keras, processed these images to predict disease stages, and its outputs were structured into CSV files using Pandas. These predictions were then merged with demographic data, cleaned by removing redundant values, and a crisp value was calculated using NumPy to quantify the prediction outcomes.

The system also included a custom-built federated learning framework, which involved manual implementation of the trained belief rule-based (BRB) model and particle swarm optimization (PSO) for parameter tuning. The federated learning setup was established by creating secure connections between client nodes and a central server using libraries like Sockets, Paramiko, and Flask. This setup allowed local models to be trained independently on different datasets and then aggregated at the server to form a global model, ensuring data privacy while improving predictive accuracy. The entire process was managed and executed in Visual Studio Code, providing a robust environment for developing and refining the Alzheimer’s disease prediction system. The code constructed for the development of the Alzheimer’s Diagnostic System is available on ERROR: Failed to execute system command: GitHub.

### 4.2. Hyperparameter Setting

The hyperparameter settings for the Federated Averaging (FedAvg) algorithm in diagnosing Alzheimer’s disease are outlined in Table 5. Initially, the CNN prediction data combined with demographic data are divided among three clients. In each client, 80% and 20% of data are available for training and testing of the optimized BRB model. For optimization of BRB, particle swarm optimization (PSO) is used. The parameters in PSO have a swarm size of 50 and a maximum of 100 iterations, with parameter bounds ranging from 0 to 1 for each of the 38 parameters. A total of five iterations is performed in PSO.

### 4.3. Evaluation Metrics

Mean square error (MSE) is a critical metric used to evaluate the performance of models in detecting Alzheimer’s disease. MSE measures the average of the squares of the errors, which are the differences between the predicted outcomes and the actual outcomes. It provides a quantitative assessment of the model’s prediction accuracy, particularly in medical diagnosis contexts such as Alzheimer’s disease detection.

(7) $$\mathrm{MSE}=\frac{1}{n}\sum_{i=1}^{n}{\left(y_{i}-{\hat{y}}_{i}\right)}^{2}$$

where:

- *n* is the number of observations or patients in the dataset.
- $y_{i}$ represents the actual diagnostic score or label for the *i*-th patient.
- ${\hat{y}}_{i}$ denotes the predicted diagnostic score or label for the *i*-th patient made by the model.

In the context of Alzheimer’s disease detection, $y_{i}$ might correspond to the true diagnostic status of a patient (e.g., Mild Cognitive Impairment, or Alzheimer’s Disease), and ${\hat{y}}_{i}$ represents the model’s prediction. The MSE metric helps to quantify how closely the model’s predictions match the true diagnostic statuses.

A lower MSE value indicates that the model’s predictions are close to the actual diagnostic results, suggesting a higher level of accuracy in detecting Alzheimer’s disease. Achieving a low MSE is crucial for ensuring the reliability of the model in clinical settings, where accurate detection of Alzheimer’s disease is essential for timely intervention and treatment.

### 4.4. Performance of CNN for MRI Classification of Alzheimer’s

**Figure 5:**
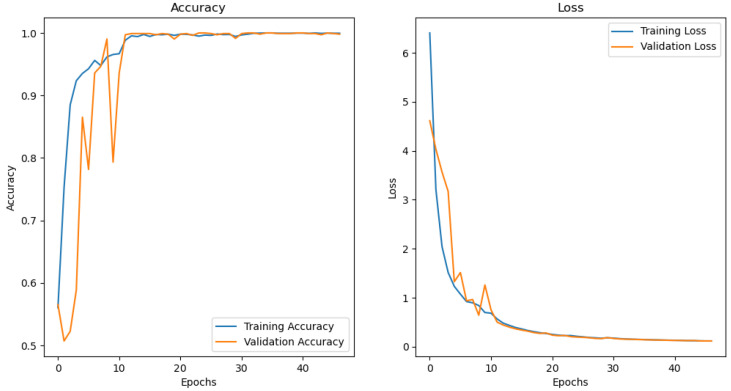
CNN performance graph.

Figure 5 illustrates the loss and accuracy graph of the customised CNN used to train and test the MRI images extracted from the ADNI dataset. It is observed that the model reached an accuracy of approximately 98% and a loss of 0.01%. It can be deduced that there is little or no difference between the training and testing accuracy and loss. This means that the model is well fit.

### 4.5. Rule Base Overview for Federated Learning Clients and Servers

The tables below represent the outcome of the rule base for the three iterations. This section is divided into two Section 4.6 and Section 4.7: one for the clients and one for the server. Section 4.6 indicates the client-side rule-base as the input rule-base which takes into account the following parameters: the CNN prediction value represented as “CNN_Value”, the age of the person represented as “Age”, and the predicted Alzheimer’s level represented as “Group”. In contrast, Section 4.7 indicates the server-side rule base as the output rule base, which takes into account the optimized values obtained after training the BRB. The optimized parameters are the likelihood of the person being diagnosed with Alzheimer’s which is distributed in three levels: Cognitively Normal “CN”, Mild Cognitive Impairment “MCI”, and Alzheimer’s Disease “AD”. To find more information about the inheritance of the rule base, refer to the initial rule base depicted in Table 4 of Section 3.4.3.

### 4.6. Rule Base for Local Training Model on Client Side

In this section, the client-side rule base from three different aggregators, namely FedAvg, FedProx, and Genetic Algorithm, are demonstrated in the following ables. Each table (Table 6, Table 7, Table 8, Table 9, Table 10, Table 11, Table 12, Table 13, Table 14, Table 15, Table 16, Table 17, Table 18, Table 19, Table 20, Table 21, Table 22, Table 23, Table 24, Table 25, Table 26, Table 27, Table 28, Table 29, Table 30, Table 31 and Table 32) illustrates the type of aggregator used, the client, and the number of iterations.

### 4.7. Optimized Rule Base for Global Training Model on Server Side

In this section, the server-side rule base from three different aggregators, namely FedAvg, FedProx, and Genetic Algorithm, are demonstrated in the following tables. Each table (Table 33, Table 34, Table 35, Table 36, Table 37, Table 38, Table 39, Table 40 and Table 41) illustrates the type of aggregator used, the client, and the number of iterations.

### 4.8. Graphical Representation of Accuracy and Loss for the Clients and Servers

In this section, some illustrations display the accuracy and loss data, for clients and servers using aggregation methods like FedAvg, Genetic Algorithm, and FedProx. These visuals present a comparison of performance measures, by depicting how local accuracy/loss changes throughout the iterations.

**Figure 6:**
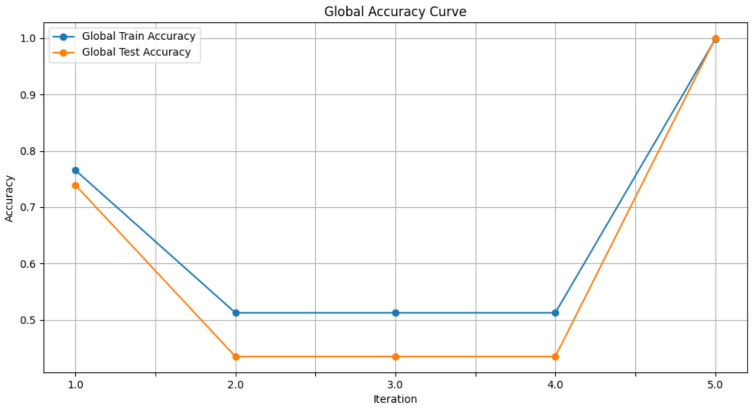
Global accuracy from FedAvg aggregator.

**Figure 7:**
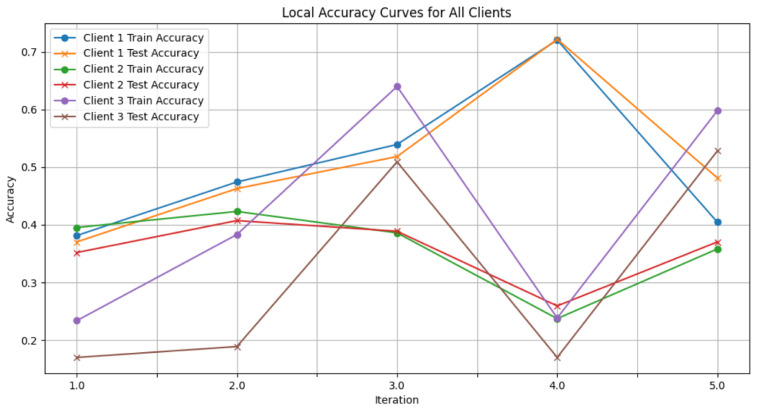
Local client accuracy from FedAvg aggregator.

**Figure 8:**
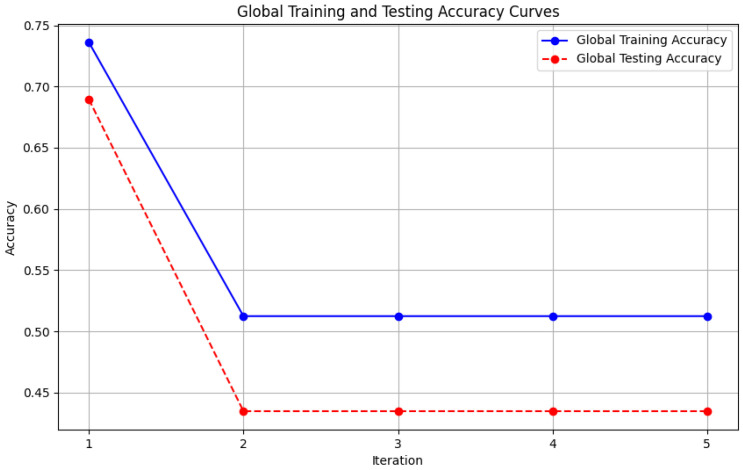
Global accuracy from Genetic Algorithm aggregator.

**Figure 9:**
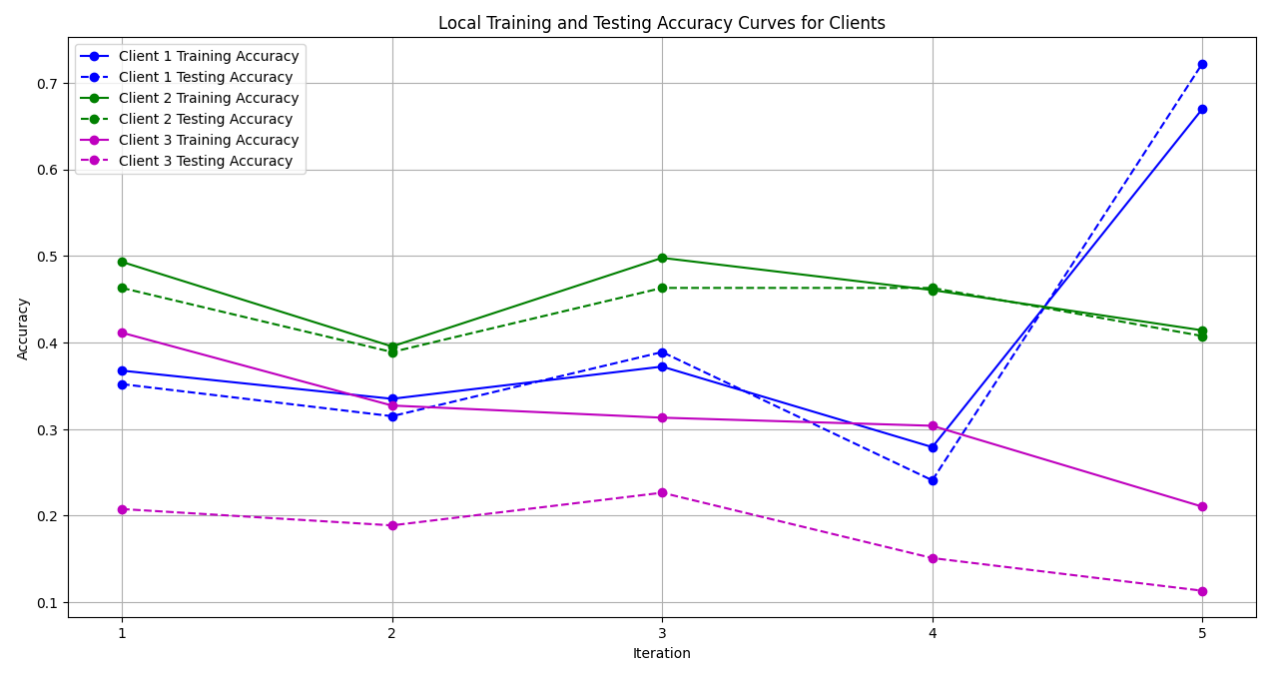
Local accuracy from Genetic Algorithm aggregator.

**Figure 10:**
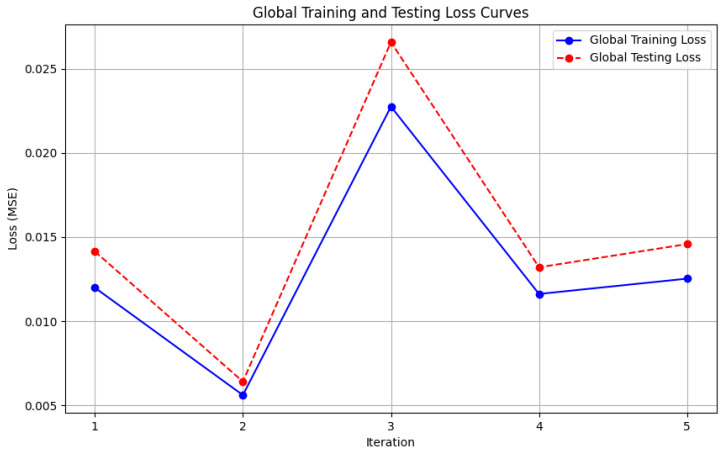
Global loss from Genetic Algorithm aggregator.

**Figure 11:**
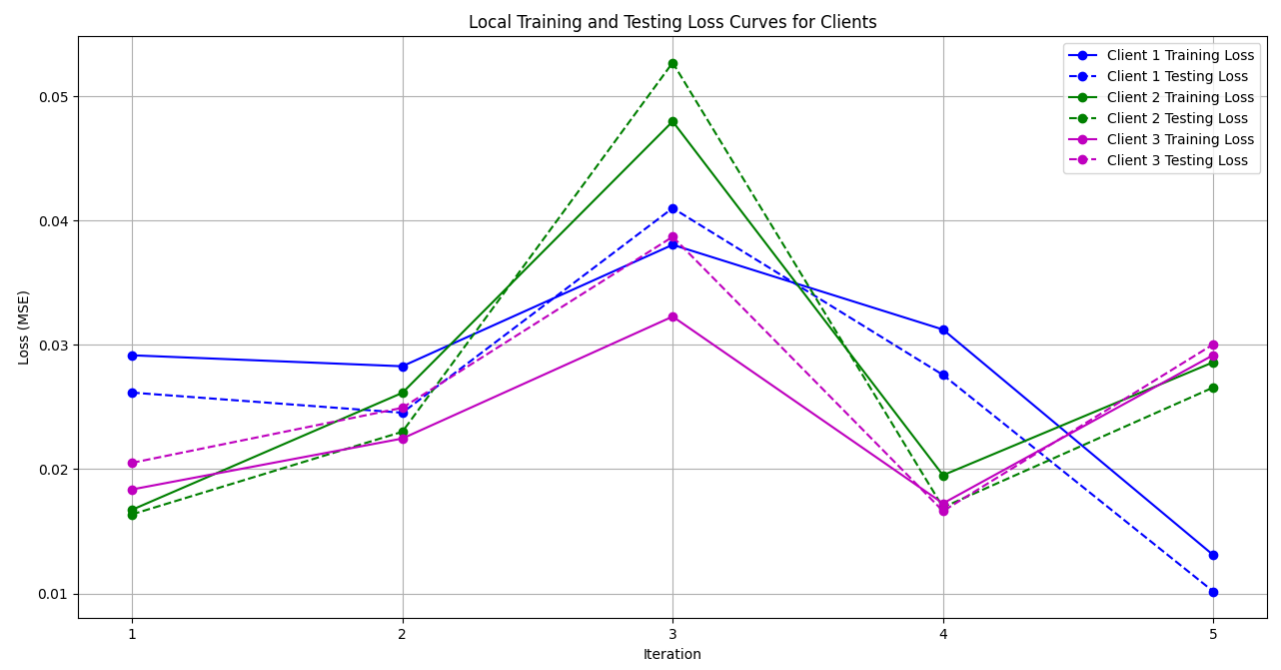
Local loss from Genetic Algorithm aggregator.

**Figure 12:**
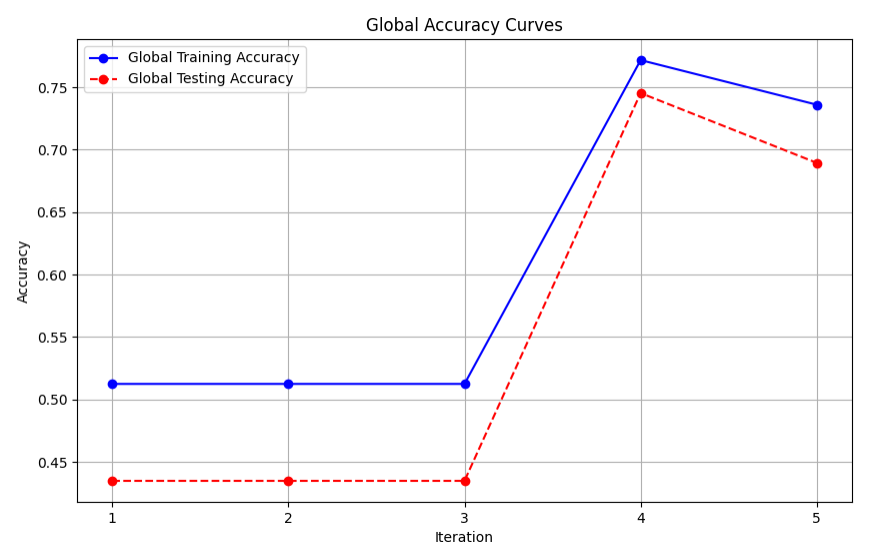
Global loss from FedProx aggregator.

**Figure 13:**
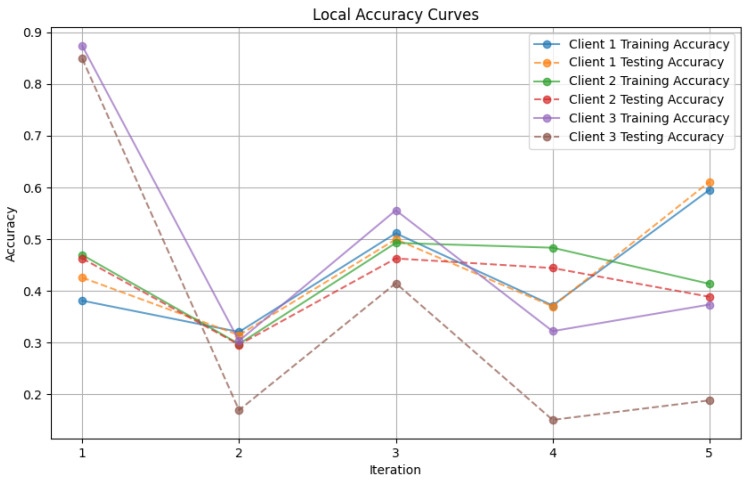
Local loss from FedProx aggregator.

- Figure 6: The graph visualizes the Global Accuracy derived using the FedAvg aggregator in the three iterations. The x-axis represents the number of iterations, and the y-axis shows the accuracy achieved during global training.
- Figure 7: This plot dictates the Local Client Accuracy from the FedAvg aggregator for each client across the three iterations. The accuracy for each client is plotted independently, demonstrating the performance consistency or variability among the clients.
- Figure 8: This figure presents the Global Accuracy achieved using the Genetic Algorithm aggregator. The plot distinguishes the training versus testing accuracy over the three iterations, illustrating the effectiveness of the Genetic Algorithm in global optimization.
- Figure 9: Similar to Figure 7, this plot shows the Local Accuracy for each client when using the Genetic Algorithm aggregator. The fluctuation in accuracy among clients across iterations is depicted.
- Figure 10: This plot depicts the Global Loss during training and testing using the Genetic Algorithm aggregator. The y-axis represents the loss, and the x-axis shows the iterations. A reduction in loss over iterations indicates better convergence.
- Figure 11: This figure shows the Local Loss for each client under the Genetic Algorithm aggregator. The figure aids in visualizing how individual clients are optimizing their loss functions.
- Figure 12: This plot shows the Global Accuracy achieved using the FedProx aggregator. It illustrates the effectiveness of FedProx in improving the global model accuracy across iterations.
- Figure 13: Finally, this plot presents the Local Accuracy for each client when using the FedProx aggregator. The comparison among clients is essential to understanding the federated learning model’s performance across distributed data sources.

These plots offer an analysis of the precision and error measurements when using approaches, in federated learning aiding in determining the best method suited to a specific situation.

### 4.9. Interpretation of Model Performance of Aggregators Used in Federated Learning

This study investigates how aggregation techniques, namely FedAvg, FedProx, and Genetic Algorithm, work within a federated learning (FL) setup to detect Alzheimer’s disease by analyzing images and demographic data. The findings detailed in Table 42 shed light on how each method performs on an overall scale.

#### 4.9.1. FedAvg Aggregator

The FedAvg aggregator, which averages the model parameters from each client, demonstrated remarkable performance. At the global level, it achieved a nearly flawless testing accuracy of 0.99999 and a minimal mean squared error (MSE) of 0.00465. This impressive performance can be attributed to several factors inherent to the FedAvg algorithm:

1. Robust Aggregation of Model Updates: The FedAvg method involves averaging the model parameters from clients to reduce the impact of data differences, among them. By blending these updates, the overall model can effectively adapt to datasets resulting in improved performance.
2. Effective Handling of Data Heterogeneity: In federated learning setups, there is often a challenge, with IID (Independent and Identically Distributed) data, where various clients possess distinct data distributions. To address this, FedAvg tackles the problem by combining updates from all clients, allowing for a representation of data patterns and minimizing the chances of tailoring to any one client’s specific data.
3. Generalization Across Multiple Clients: The variety of data from clients helps the overall model perform effectively in situations. This is shown by the testing accuracy of the model, which is close to 1.0, proving its excellent performance, with different types of data inputs.

Despite its performance, there are clear differences, in accuracy levels across different clients. For instance, client 3 only managed to achieve a testing accuracy of 0.52830, underscoring the difficulties FedAvg may face in adapting to client nuances. This disparity indicates that, while FedAvg excels in building a model, it might struggle to account for the distinct traits present in individual client datasets. Moreover, from Table 43, it can be deduced that the average time required to run PSO is 7,721.84 s. Now, if the number of iteration alongside rules increases, the time required to run BRBs will also increase. So, this raises an issue of time complexity which will not be applicable for real-time applications which support large-scale datasets with large rule sets.

#### 4.9.2. FedProx Aggregator

FedProx, a modified version of FedAvg that incorporates a term to address data differences efficiently, also exhibited impressive results. It reached a testing accuracy of 0.68944 with an MSE of 0.00687. The inclusion of the term in FedProx contributes to enhancing the training process in scenarios with diverse data.

The consistent performance outcomes among clients using FedProx imply that its regularization features offer benefits in maintaining performance stability across varying data distributions. For example, client 1 achieved a testing accuracy of 0.61111, notably surpassing the results seen with FedAvg, suggesting that FedProx might excel in managing types of data disparities.

#### 4.9.3. Genetic Algorithm Aggregator

The Genetic Algorithm (GA) aggregator, which evolves model parameters based on a fitness function, demonstrated potential in adapting to client-specific data. For example, client 1 achieved a testing accuracy of 0.29629 with an MSE of 0.032451. However, the global accuracy was lower, with a testing accuracy of 0.43478 and an MSE of 0.00904.

The lower global accuracy implies that, while GA is adept at fine-tuning models for individual clients, it may require more extensive fine-tuning or a larger population size to reach the same level of generalization as FedAvg. Nonetheless, the adaptability of GA highlights its potential for scenarios wherein client data vary significantly, suggesting that, with further refinement, GA could become a more robust aggregator in FL settings.

### 4.10. Comparison with State-of-the-Art Methods

In times, there have been advancements in methods to improve the accuracy and reliability of diagnosing Alzheimer’s disease (see Table 44). These cutting-edge techniques leverage sophisticated machine learning models. However, our proposed method shows progress in accuracy and maintaining privacy setting, a benchmark in this field.

For example, Umme [1] and colleagues utilized DementiaNet with transfer learning to achieve a 97% accuracy on an MRI dataset with 6400 images. While this technique enhances performance on medical imaging tasks using trained models, it falls short in addressing data privacy concerns and uncertainties. Similarly, Raees et al. [40] combined support vector machines (SVMs) with deep neural networks (DNNs) to analyze data from 111 individuals in an MCI dataset achieving a 90% accuracy rate. While effective for datasets, this approach may require modifications to generalize to larger and more complex datasets. Buvaneswari et al. [41] experimented with SegNet and ResNet 101 on the ADNI dataset obtaining an accuracy of 96%. This method makes use of learning for feature extraction and segmentation, proving particularly effective for datasets like ADNI. However, there are still challenges when it comes to maintaining data privacy and handling uncertainties in the predictions. In a study by Saratxaga et al. [42] (2021), they created a model using ResNet 18 and BrainNet utilizing datasets from Kaggle. They found varying levels of accuracy ranging from 80% to 90% depending on the dataset used. While their method shows promise, the fluctuations in accuracy indicate a need for improvements to ensure consistency across datasets. Another study by Hu et al. [43] (2021) employed a convolutional neural network (CNN) trained on ADNI and NIFD datasets achieving an accuracy of 92%. CNNs are known for their effectiveness in image classification tasks as they can automatically learn features from raw image data. However, similar to other approaches, this method lacks mechanisms for safeguarding data privacy during the training process. Khalil et al. [5] (2023) investigated the use of hardware acceleration with VHDL and FPGA for diagnosing Alzheimer’s disease using simulation data. Despite reaching an accuracy of 89%, with a sensitivity rate of 87% and low power consumption (35–39 mW), this technique primarily emphasizes hardware efficiency rather than enhancing diagnostic accuracy or addressing concerns regarding data privacy. In their work, Mitrovska et al. [6] (2024) introduced a federated learning framework integrated with aggregation (SecAgg) and demographic simulations. The researchers focus on the importance of ensuring privacy in their work and analyze variations although they do not provide accuracy metrics. This underscores the increasing awareness of privacy in handling data. This also suggests that their method may need further refinement to achieve precise diagnostic results. In their study, Trivedi et al. [32] and colleagues applied federated deep learning using AlexNet on a distributed dataset achieving an accuracy rate of 98.53%. Their research stands out for its emphasis on safeguarding data privacy and demonstrating reliability across both multiple client settings. While this approach marks progress in learning, there is still room for enhancing the management of data distributions and addressing uncertainties. Altalbe et al. [33] developed a network within a federated learning framework for analyzing Kay Elemetrics voice disorder data reaching an accuracy level of 82.82%. Their strategy concentrates on mitigating overfitting and upholding privacy standards for medical applications. However, there is potential for enhancing accuracy, particularly when dealing with more varied datasets. Mandawkar et al. [34] utilized the Tawny Flamingo Deep CNN model on datasets achieving accuracies of 98.252% through Kfold validation and 97.995% during training sessions. Their methodology showcases the effectiveness of adjustments in attaining precision results; however, it falls short in addressing privacy concerns or uncertainties. Castro et al. [35] merged federated learning with biometric authentication and tested this on the OASIS and ADNI datasets. They found accuracy levels focused primarily on safeguarding privacy and preventing data manipulation, both essential aspects in federated learning settings.This strategy highlights the balance between security and performance, although there is room for enhancements in accuracy to improve its practical utility. On a similar note, Qian et al. [9] introduced FeDeFo, a combination of deep forest with federated learning applied to sMRI images. Their approach prioritizes model development while ensuring data privacy even though specific accuracy metrics were not disclosed. This indicates an emphasis on privacy protection and customized modeling, suggesting advancements in accuracy down the line.

In contrast, our research proposes a hybrid deep and federated learning methodology implemented on the ADNI and NIfTI files dataset. This approach achieved a 99.9% accuracy rate, significantly surpassing existing methods’ reported accuracies. Moreover, our model prioritizes data privacy and effectively manages uncertainties, offering a solution to the challenges associated with diagnosing Alzheimer’s disease. By combining the strengths of learning with the benefits of federated learning, our model not only establishes a new standard for diagnostic precision, but also addresses crucial concerns regarding data privacy and uncertainty management. This makes it a robust and dependable tool for world applications.

## 5. Conclusions and Future Research Directions

In this study, a method is proposed for diagnosing Alzheimer’s disease, addressing challenges such as data variability, privacy concerns, and uncertainty in datasets. CNNs analyze MRI scans, while federated learning ensures patient data privacy, and the BRB system manages diagnostic uncertainty. Experiments evaluating FedAvg, FedProx, and Genetic Algorithm aggregation methods revealed that FedAvg achieved near-perfect accuracy (99.99%) and minimal mean squared error (MSE), demonstrating strong generalization across datasets but limited performance for client-specific variations. FedProx delivered consistent results by effectively addressing client-specific data differences through regularization, while the Genetic Algorithm showed adaptability to diverse data distributions, requiring additional fine-tuning but highlighting its potential for optimizing client-specific parameters.

The proposed framework applies convolutional neural networks (CNNs) in Alzheimer’s disease classification. This heavily relies on access to labeled datasets. Even with the use of techniques like data augmentation and transfer learning, there is still a challenge in ensuring the model’s adaptability to unseen data.

In order to overcome this limitation, the FL framework was employed. This framework is beneficial in sensitive domains such as healthcare. Because it ensures that patient data remain localized on client devices, FL also minimizes the risk of privacy breaches. In the FL approach, these advantages are further enhanced by optimizing the model’s performance across heterogeneous client datasets to enable robust training under uncertainty without compromising data privacy.

However, it is important to recognize the limitations of this research. The implementation of federated learning (FL) which plays a role in safeguarding data privacy, has brought about challenges concerning communication model convergence. In scenarios wherein datasets vary widely, maintaining model performance across nodes has proven to be quite challenging. Despite the utilization of strategies like model averaging and communication-efficient algorithms, there is still a need for optimization within FL frameworks.

Regarding the belief rule base (BRB) system, known for its effectiveness in handling uncertainty, it poses complexity concerns, especially when combined with particle swarm optimization (PSO). This complexity could potentially hinder large-scale applications in real-time situations that require decision-making.

Several avenues for exploration are proposed for future to improve the efficiency and applicability of the framework in real-life situations.

To optimize training and inference times, hardware acceleration tools should be explored to address the complexity of federated learning and belief rule-based systems. These tools can significantly reduce computational overhead, making the framework more feasible for resource-constrained environments. Enhanced data security measures, such as privacy-preserving techniques and homomorphic encryption, should also be integrated to safeguard patient information beyond the privacy provided by federated learning.

Testing the scalability of the framework with diverse datasets is essential, along with extending its application to other neurodegenerative diseases or medical conditions requiring privacy and uncertainty management. This would highlight the framework’s adaptability and robustness across various healthcare contexts.

Practical considerations for large-scale deployment, including resource requirements, must be addressed. Collaborations with healthcare providers and regulatory agencies will be essential to ensure the system meets high diagnostic standards and integrates seamlessly into existing healthcare processes.

Future research should focus on incorporating additional data modalities, such as genetic information, lifestyle factors, and longitudinal data, to improve the precision of the diagnosis of Alzheimer’s disease. Expanding data sources could improve the model’s ability to detect patterns and relationships, enabling more precise and comprehensive diagnoses. Trials in real-world settings will be crucial to validate the framework’s efficacy, with collaboration from healthcare institutions to deploy and monitor its performance while gathering feedback from experts. Moreover, future work should explore cross-dataset performance evaluation to enhance generalization and extend the framework’s applicability across broader medical domains.

## Figures and Tables

**Table 1:** Summary of key studies in CNN-based Alzheimer’s diagnosis.

Study	Data Type	Methodology	Accuracy	Key Findings	Limitations
Marwa et al. [19]	MRI Images	DNN	99.68%	High accuracy for various stages	Limited generalizability
Ghazal et al. [20]	MRI Images	Transfer Learning (AlexNet)	91.7%	Effective for stage classification	Potential overfitting
AlSaeed et al. [21]	MRI Images	CNN (ResNet50)	85.7–99%	Superior performance in feature extraction	Not evaluated on diverse datasets
Helaly et al. [23]	MRI Images	CNN, Transfer Learning	97%	VGG-19 model performed well in classification	Limited comparison with other DL models
Mohammed et al. [24]	MRI Images	CNN, Hybrid Models	94.8%	Hybrid models improved diagnostic accuracy	Requires large datasets for training
Ji et al. [30]	MRI Images	Ensemble Learning	97.65%	Early diagnosis of AD/mild cognitive impairment	Ensemble methods can be computationally expensive
Islam et al. [31]	MRI Images	CNN	Not Specified	Fast AD detection suitable for small datasets	Not detailed in feature extraction

**Table 2:** Key summary of studies in Alzheimer’s disease detection using federated learning.

Study	Data Type	Methodology	Accuracy	Key Findings	Limitations
Khalil et al. [5]	MRI Images, Simulation Data	Federated Learning (VHDL + FPGA)	89%	Efficient hardware acceleration for FL with low power consumption	Limited to specific hardware settings
Mitrovska et al. [6]	Demographic and Diagnosis Data	Federated Averaging (FedAvg) + Secure Aggregation (SecAgg)	Not Specified	Enhanced privacy guarantees with FL; effective under diverse demographics	Potential complexity in real-world deployment
Trivedi et al. [32]	MRI Images	Federated Deep Learning (AlexNet)	98.53%	High accuracy in AD classification with privacy-preserving setup	Requires IID datasets
Altalbe et al. [33]	Voice Disorder Data	Federated Learning (DNN)	82.82%	Overfitting reduced through iterative training; good privacy preservation	Lower accuracy compared to other models
Mandawkar et al. [34]	Clinical Data	Tawny Flamingo Deep CNN + Federated Learning	97.995%	High accuracy in AD detection; effective in distributed settings	Computationally intensive
Castro et al. [35]	RGB MRI Images	Federated Learning + Biometric Recognition	Comparable to Centralized ML	Privacy preservation in AD diagnosis with biometric authentication	Performance depends on biometric accuracy
Qian et al. [9]	sMRI Images	Federated Deep Forest (FeDeFo)	Not Specified	Personalized AD classification; effective handling of data discrepancies	Complexity in model personalization

**Table 3:** Modified CNN model architecture.

Model Content	Details
1st Convolution Layer 2D	Input Size 128 × 128 × 3, 16 filters of kernel size 3 × 3, he_normal initializer, L2 (0.01), LeakyReLU (alpha = 0.001)
1st Max Pooling Layer 2D	Pool size 2 × 2
Batch Normalization	Normalizes activations
2nd Convolution Layer 2D	32 filters of kernel size 3 × 3, he_normal initializer, L2 (0.01), LeakyReLU (alpha = 0.001)
2nd Max Pooling Layer 2D	Pool size 2 × 2
3rd Convolution Layer 2D	128 filters of kernel size 3 × 3, he_normal initializer, L2 (0.01), LeakyReLU (alpha = 0.001)
3rd Max Pooling Layer 2D	Pool size 2 × 2
Batch Normalization	Normalizes activations
Flattening Layer	Converts 2D matrix to 1D vector
Dense Layer	128 units, he_normal initializer, L2 (0.01), LeakyReLU (alpha = 0.001)
Dropout Layer	Randomly deactivates 25% neurons
Dense Layer	64 units, LeakyReLU (alpha = 0.001)
Dropout Layer	Randomly deactivates 25% neurons
Output Layer	4 units, SoftMax activation
Optimization Function	Adam optimizer
Loss Function	Sparse Categorical Crossentropy
Metrics	Accuracy

**Table 4:** Initial rule base.

Rule ID	Rule Weight	IF		THEN Group	Activation Weight
		CNN_Value	Age		
R1	1.0	Low	Young-Old	CN	0.36
R2	1.0	Low	Middle-Old	MCI	0.40
R3	1.0	Low	Old-Old	MCI	0.22
R4	1.0	Medium	Young-Old	MCI	0.18
R5	1.0	Medium	Middle-Old	AD	0.45
R6	1.0	Medium	Old-Old	AD	0.30
R7	1.0	High	Young-Old	AD	0.55
R8	1.0	High	Middle-Old	AD	0.65
R9	1.0	High	Old-Old	AD	0.80

**Table 5:** Hyperparameter setting for FedAvg.

Hyperparameter	Value
Train-test split	80% training, 20% testing
Scaling method	MinMaxScaler
PSO parameters:	Swarm size: 50 Maximum iterations: 100 Lower bounds: [0, 0, …, 0] (38 times) Upper bounds: [1, 1, …, 1] (38 times)
Number of clients	3
Iterations for federated learning	5

**Table 6:** Rule base for FedAvg aggregator client 1: first iteration.

Rule ID	Rule Weight	CNN_Value	Age	Group
R1	0.0	0.1656	0.7499	0.0845
R2	0.7123	0.7404	0.2001	0.0595
R3	0.0	0.4954	0.2110	0.2936
R4	0.7882	0.0072	0.2619	0.7309
R5	0.9821	0.6643	0.0000	0.3357
R6	0.9650	0.5683	0.0000	0.4317
R7	0.9971	0.0777	0.0212	0.9011
R8	0.4432	0.3439	0.6561	0.0000
R9	0.0542	0.0000	0.7270	0.2730

**Table 7:** Rule base for FedAvg aggregator client 2: first iteration.

Rule ID	Rule Weight	CNN_Value	Age	Group
R1	0.7512	0.0026	0.6759	0.3215
R2	0.5443	0.0000	0.5141	0.4859
R3	0.0666	0.0002	0.6458	0.3540
R4	0.5061	0.6815	0.3185	0.0000
R5	0.8389	0.7446	0.2265	0.0289
R6	0.3314	0.9831	0.0169	0.0000
R7	1.0000	0.0059	0.3432	0.6510
R8	0.0023	0.0014	0.4648	0.5338
R9	0.1576	0.2998	0.1973	0.5029

**Table 8:** Rule base for FedAvg aggregator client 3: first iteration.

Rule ID	Rule Weight	CNN_Value	Age	Group
R1	0.6234	0.5289	0.0000	0.4711
R2	0.1875	0.5248	0.2184	0.2568
R3	1.0000	1.0000	0.0000	0.0000
R4	1.0000	0.2385	0.0177	0.7437
R5	0.3468	0.4117	0.4524	0.1359
R6	0.9086	0.0001	0.9976	0.0023
R7	1.0000	0.0056	0.3551	0.6393
R8	0.6326	0.0000	0.4097	0.5903
R9	0.9736	0.5891	0.3160	0.0949

**Table 9:** Rule base for FedAvg aggregator client 1: second iteration.

Rule ID	Rule Weight	CNN_Value	Age	Group
R1	0.2869	0.7223	0.2777	0.0000
R2	0.6418	0.7559	0.1218	0.1223
R3	0.4251	0.1436	0.3681	0.4883
R4	0.9871	0.0046	0.3004	0.6950
R5	0.4296	0.0000	0.4848	0.5152
R6	0.0022	0.2094	0.0000	0.7906
R7	0.3891	0.2848	0.3085	0.4067
R8	0.4943	0.4355	0.0000	0.5645
R9	0.4881	0.4510	0.3779	0.1711

**Table 10:** Rule base for FedAvg aggregator client 2: second iteration.

Rule ID	Rule Weight	CNN_Value	Age	Group
R1	0.0000	0.4824	0.4045	0.1132
R2	0.2866	0.0000	0.7747	0.2253
R3	0.8641	0.0842	0.9158	0.0000
R4	0.0007	0.7054	0.2771	0.0175
R5	0.4554	0.3732	0.6268	0.0000
R6	0.2630	0.6074	0.0000	0.3926
R7	0.5805	0.8676	0.0346	0.0978
R8	1.0000	0.0041	0.2227	0.7731
R9	0.1764	0.0006	0.3066	0.6928

**Table 11:** Rule base for FedAvg aggregator client 3: second iteration.

Rule ID	Rule Weight	CNN_Value	Age	Group
R1	0.0907	0.5785	0.3410	0.0805
R2	0.6633	0.0039	0.2294	0.7666
R3	0.3330	0.0841	0.0003	0.9156
R4	0.0000	0.4099	0.3201	0.2700
R5	0.4140	0.0090	0.2667	0.7243
R6	0.6652	0.0003	0.6918	0.3079
R7	0.9462	0.9070	0.0295	0.0634
R8	0.9036	0.4595	0.0000	0.5405
R9	0.0000	0.4054	0.1314	0.4632

**Table 12:** Rule base for FedAvg aggregator client 1: third iteration.

Rule ID	Rule Weight	CNN_Value	Age	Group
R1	0.9997	0.0017	0.6385	0.3598
R2	0.4324	0.4962	0.5038	0.0000
R3	0.5750	0.7245	0.0000	0.2755
R4	0.8920	0.1016	0.0050	0.8934
R5	0.1937	0.7326	0.1164	0.1509
R6	0.2851	0.0001	0.4976	0.5023
R7	0.0632	0.8496	0.1204	0.0300
R8	0.9109	0.7154	0.0000	0.2846
R9	0.0000	0.6565	0.1659	0.1776

**Table 13:** Rule base for FedAvg aggregator client 2: third iteration.

Rule ID	Rule Weight	CNN_Value	Age	Group
R1	0.0907	0.5785	0.3410	0.0805
R2	0.6633	0.0039	0.2294	0.7666
R3	0.3330	0.0842	0.0003	0.9156
R4	0.0000	0.4099	0.3201	0.2700
R5	0.4140	0.0090	0.2667	0.7243
R6	0.6652	0.0003	0.6918	0.3079
R7	0.9462	0.9070	0.0295	0.0634
R8	0.9036	0.4595	0.0000	0.5405
R9	0.0000	0.4054	0.1314	0.4632

**Table 14:** Rule base for FedAvg aggregator client 3: third iteration.

Rule ID	Rule Weight	CNN_Value	Age	Group
R1	0.9959	0.0000	0.6876	0.3124
R2	0.5408	0.5597	0.0000	0.4403
R3	0.0000	0.3782	0.2889	0.3329
R4	1.0000	0.0728	0.0007	0.9265
R5	0.0863	0.7150	0.0405	0.2447
R6	0.7342	0.1596	0.0000	0.8404
R7	0.0796	0.0000	0.4875	0.5125
R8	0.0001	0.5486	0.4514	0.0000
R9	0.6853	0.0000	0.3189	0.6810

**Table 15:** FedProx aggregator client 1 rule base: first iteration.

Rule ID	Rule Weight	CNN Value	Age	Group
R1	0.0956	0.3251	0.5028	0.1721
R2	0.2457	0.6778	0.0000	0.3222
R3	0.7022	0.0000	0.6496	0.3504
R4	0.9938	0.7386	0.2160	0.0453
R5	0.7375	0.0004	0.6380	0.3616
R6	0.0011	0.0487	0.0742	0.8771
R7	0.7026	0.0000	0.8207	0.1793
R8	0.1263	0.0412	0.9588	0.0000
R9	1.0000	0.7382	0.0000	0.2618

**Table 16:** FedProx aggregator client 2 rule base: first iteration.

Rule ID	Rule Weight	CNN Value	Age	Group
R1	0.0000	0.3251	0.5028	0.1721
R2	0.2457	0.6778	0.0000	0.3222
R3	0.7022	0.0000	0.6496	0.3504
R4	0.9938	0.7386	0.2160	0.0453
R5	0.7375	0.0004	0.6380	0.3616
R6	0.0011	0.0487	0.0742	0.8771
R7	0.7026	0.0000	0.8207	0.1793
R8	0.1263	0.0412	0.9588	0.0000
R9	1.0000	0.7382	0.0000	0.2618

**Table 17:** FedProx aggregator client 3 rule base: first iteration.

Rule ID	Rule Weight	CNN Value	Age	Group
R1	0.0000	0.3251	0.5028	0.1721
R2	0.2457	0.6778	0.0000	0.3222
R3	0.7022	0.0000	0.6496	0.3504
R4	0.9938	0.7386	0.2160	0.0453
R5	0.7375	0.0004	0.6380	0.3616
R6	0.0011	0.0487	0.0742	0.8771
R7	0.7026	0.0000	0.8207	0.1793
R8	0.1263	0.0412	0.9588	0.0000
R9	1.0000	0.7382	0.0000	0.2618

**Table 18:** FedProx aggregator client 1 rule base: second iteration.

Rule ID	Rule Weight	CNN Value	Age	Group
R1	0.0000	0.2123	0.7077	0.0800
R2	0.8953	0.0000	0.7188	0.2812
R3	0.4688	0.4743	0.5055	0.0201
R4	0.4004	0.3913	0.2215	0.3872
R5	0.5339	0.0001	0.3351	0.6648
R6	0.4359	0.8704	0.0903	0.0393
R7	0.9764	0.6474	0.0510	0.3016
R8	0.8233	0.0005	0.0981	0.9015
R9	0.0301	0.4195	0.5728	0.0077

**Table 19:** FedProx aggregator client 2 rule base: second iteration.

Rule ID	Rule Weight	CNN Value	Age	Group
R1	0.0000	0.2123	0.7077	0.0800
R2	0.8953	0.0000	0.7188	0.2812
R3	0.4688	0.4743	0.5055	0.0201
R4	0.4004	0.3913	0.2215	0.3872
R5	0.5339	0.0001	0.3351	0.6648
R6	0.4359	0.8704	0.0903	0.0393
R7	0.9764	0.6474	0.0510	0.3016
R8	0.8233	0.0005	0.0981	0.9015
R9	0.0301	0.4195	0.5728	0.0077

**Table 20:** FedProx aggregator client 3 rule base: second iteration.

Rule ID	Rule Weight	CNN Value	Age	Group
R1	0.0000	0.2123	0.7077	0.0800
R2	0.8953	0.0000	0.7188	0.2812
R3	0.4688	0.4743	0.5055	0.0201
R4	0.4004	0.3913	0.2215	0.3872
R5	0.5339	0.0001	0.3351	0.6648
R6	0.4359	0.8704	0.0903	0.0393
R7	0.9764	0.6474	0.0510	0.3016
R8	0.8233	0.0005	0.0981	0.9015
R9	0.0301	0.4195	0.5728	0.0077

**Table 21:** FedProx aggregator client 1 rule base: third iteration.

Rule ID	Rule Weight	CNN Value	Age	Group
R1	0.0489	0.0000	0.1579	0.8421
R2	0.9898	0.7954	0.0000	0.2046
R3	0.2992	0.2640	0.7360	0.0000
R4	0.5001	0.6976	0.0000	0.3024
R5	0.9721	0.4644	0.0000	0.5356
R6	0.5106	0.0000	0.7867	0.2133
R7	1.0000	0.0016	0.5032	0.4952
R8	0.6459	0.7756	0.2244	0.0000
R9	0.7654	0.6747	0.0183	0.3070

**Table 22:** FedProx aggregator client 2 rule base: third iteration.

Rule ID	Rule Weight	CNN Value	Age	Group
R1	0.0489	0.0000	0.1579	0.8421
R2	0.9898	0.7954	0.0000	0.2046
R3	0.2992	0.2640	0.7360	0.0000
R4	0.5001	0.6976	0.0000	0.3024
R5	0.9721	0.4644	0.0000	0.5356
R6	0.5106	0.0000	0.7867	0.2133
R7	1.0000	0.0016	0.5032	0.4952
R8	0.6459	0.7756	0.2244	0.0000
R9	0.7654	0.6747	0.0183	0.3070

**Table 23:** FedProx aggregator client 3 rule base: third iteration.

Rule ID	Rule Weight	CNN Value	Age	Group
R1	0.0489	0.0000	0.1579	0.8421
R2	0.9898	0.7954	0.0000	0.2046
R3	0.2992	0.2640	0.7360	0.0000
R4	0.5001	0.6976	0.0000	0.3024
R5	0.9721	0.4644	0.0000	0.5356
R6	0.5106	0.0000	0.7867	0.2133
R7	1.0000	0.0016	0.5032	0.4952
R8	0.6459	0.7756	0.2244	0.0000
R9	0.7654	0.6747	0.0183	0.3070

**Table 24:** Rule base for Genetic Algorithm aggregator client 1: first iteration.

Rule ID	Rule Weight	CNN Value	Age	Group
R1	0.0000	0.6403	0.0000	0.3597
R2	0.9376	0.2420	0.0000	0.7580
R3	0.0000	0.0775	0.0506	0.8719
R4	0.7595	0.1560	0.0007	0.8433
R5	0.5355	0.8803	0.0056	0.1141
R6	0.5329	0.0833	0.9167	0.0000
R7	0.6066	0.0006	0.5928	0.4065
R8	0.3410	0.5007	0.4993	0.0000
R9	0.3354	0.0003	0.1079	0.8918

**Table 25:** Rule base for Genetic Algorithm aggregator client 2: first iteration.

Rule ID	Rule Weight	CNN Value	Age	Group
R1	0.0000	0.0916	0.0789	0.8295
R2	1.0000	0.5924	0.0000	0.4076
R3	0.0064	0.0000	0.6615	0.3385
R4	0.8879	0.0006	0.1202	0.8792
R5	0.2335	0.6908	0.3092	0.0000
R6	0.8953	0.0002	0.1252	0.8746
R7	0.4955	0.0025	0.4704	0.5271
R8	1.0000	0.6585	0.3393	0.0023
R9	0.7271	0.6995	0.0000	0.3005

**Table 26:** Rule base for Genetic Algorithm aggregator client 3: first iteration.

Rule ID	Rule Weight	CNN Value	Age	Group
R1	0.2760	0.6181	0.0000	0.3819
R2	0.6066	0.0002	0.0000	0.9998
R3	0.0000	0.1505	0.5868	0.2627
R4	0.3360	0.0000	0.1487	0.8513
R5	0.0648	0.2981	0.7019	0.0000
R6	0.8948	0.0000	0.6912	0.3088
R7	0.5753	0.4126	0.5874	0.0000
R8	0.0295	0.5479	0.4335	0.0187
R9	0.2985	0.5549	0.0000	0.4451

**Table 27:** Rule base for Genetic Algorithm aggregator client 1: second iteration.

Rule ID	Rule Weight	CNN Value	Age	Group
R1	0.0000	0.2409	0.6108	0.1483
R2	0.9916	0.0000	0.8724	0.1276
R3	0.9982	0.0002	0.9226	0.0772
R4	0.4423	0.0002	0.9988	0.0011
R5	0.9752	0.8539	0.0000	0.1461
R6	0.3286	0.4222	0.5778	0.0000
R7	0.8676	0.9455	0.0442	0.0103
R8	0.9292	0.0018	0.1299	0.8683
R9	0.2586	0.7650	0.0000	0.2349

**Table 28:** Rule base for Genetic Algorithm aggregator client 2: second iteration.

Rule ID	Rule Weight	CNN Value	Age	Group
R1	0.7287	0.0010	0.9990	0.0000
R2	1.0000	0.0000	0.4366	0.5634
R3	1.0000	0.0000	1.0000	0.0000
R4	0.4041	0.0000	0.8518	0.1482
R5	0.1697	0.0000	0.0000	1.0000
R6	0.7189	0.3120	0.0000	0.6880
R7	0.0050	0.4548	0.3923	0.1529
R8	0.5496	0.0000	0.0269	0.9731
R9	0.0409	0.6471	0.0000	0.3529

**Table 29:** Rule base for Genetic Algorithm aggregator client 3: second iteration.

Rule ID	Rule Weight	CNN Value	Age	Group
R1	0.6438	0.4091	0.5909	0.0000
R2	0.6435	0.0009	0.5823	0.4168
R3	0.4093	0.0000	0.8276	0.1724
R4	0.7355	0.0805	0.0000	0.9195
R5	0.4807	0.0011	0.3556	0.6434
R6	0.8471	0.6813	0.3187	0.0000
R7	0.8134	0.5222	0.3630	0.1147
R8	1.0000	0.0009	0.9573	0.0422
R9	0.6295	1.0000	0.0000	0.0000

**Table 30:** Rule base for Genetic Algorithm aggregator client 1: third iteration.

Rule ID	Rule Weight	CNN Value	Age	Group
R1	0.0259	0.0000	0.4371	0.5629
R2	0.9548	0.0005	0.1798	0.8202
R3	0.2521	0.0175	0.9825	0.0000
R4	0.6134	0.8146	0.0064	0.1790
R5	0.0410	0.0006	0.5337	0.4657
R6	0.7805	0.0001	0.1645	0.8354
R7	0.0000	0.5033	0.0631	0.4336
R8	0.7242	0.1037	0.8963	0.0000
R9	0.3425	0.4387	0.0000	0.5613

**Table 31:** Rule base for Genetic Algorithm aggregator client 2: third iteration.

Rule ID	Rule Weight	CNN Value	Age	Group
R1	0.9841	0.7833	0.0378	0.1799
R2	0.0186	0.2679	0.3714	0.3608
R3	0.4292	0.1414	0.6634	0.1951
R4	0.6449	0.0905	0.0000	0.9095
R5	0.5903	0.0049	0.9838	0.0113
R6	0.0000	0.0008	0.1789	0.8204
R7	0.0808	0.6489	0.2400	0.1112
R8	1.0000	0.0085	0.2246	0.7669
R9	0.7350	0.1810	0.0010	0.8180

**Table 32:** Rule base for Genetic Algorithm aggregator client 3: third iteration.

Rule ID	Rule Weight	CNN Value	Age	Group
R1	0.5367	0.8086	0.0000	0.1914
R2	0.4920	0.0005	0.2176	0.7818
R3	0.2167	0.0002	0.1888	0.8111
R4	0.3923	0.6196	0.3804	0.0000
R5	0.9993	0.0051	0.2194	0.7755
R6	0.0000	0.3821	0.2385	0.3794
R7	0.3796	0.6376	0.2802	0.0822
R8	0.2942	0.0012	0.2127	0.7861
R9	0.0000	0.2058	0.0277	0.7665

**Table 33:** Optimized server for FedAvg aggregator rule base: first iteration.

Rule ID	Rule Weight	CN	MCI	AD
R1	0.9612	0.0000	0.9689	0.0311
R2	0.3388	0.0000	0.4785	0.5215
R3	0.0850	0.8647	0.0747	0.0606
R4	0.0806	0.4947	0.5053	0.0000
R5	0.3647	0.0000	0.8442	0.1558
R6	0.4242	0.0000	0.0772	0.9228
R7	0.4484	0.7169	0.2821	0.0010
R8	0.0000	0.0000	0.6721	0.3279
R9	0.6411	0.0000	1.0000	0.0000

**Table 34:** Final optimized server for FedAvg aggregator rule base: second iteration.

Rule ID	Rule Weight	CN	MCI	AD
R1	0.9997	0.0758	0.0000	0.9242
R2	0.2077	0.8508	0.1492	0.0000
R3	0.3042	0.3163	0.6837	0.0000
R4	0.2439	0.5159	0.0941	0.3899
R5	0.5673	0.0007	0.6432	0.3561
R6	0.8671	0.6333	0.2925	0.0742
R7	0.9215	0.5557	0.4443	0.0000
R8	0.5669	0.0033	0.0942	0.9025
R9	0.7453	0.2629	0.7371	0.0000

**Table 35:** Optimized server for FedAvg aggregator rule base: third iteration.

Rule ID	Rule Weight	CN	MCI	AD
R1	0.3629	0.4477	0.0000	0.5523
R2	0.0000	0.2364	0.5648	0.1988
R3	0.0000	0.2970	0.3287	0.3742
R4	0.7316	0.4522	0.5478	0.0000
R5	0.1970	0.5290	0.4710	0.0000
R6	0.6037	0.6760	0.3241	0.0000
R7	0.2779	0.0001	0.1816	0.8183
R8	0.5331	0.9031	0.0449	0.0521
R9	0.2306	0.6962	0.0000	0.3038

**Table 36:** Optimized server for FedProx aggregator rule base: first iteration.

Rule ID	Rule Weight	CN	MCI	AD
R1	0.0956	0.3251	0.5028	0.1721
R2	0.2457	0.6778	0.0000	0.3222
R3	0.7022	0.0000	0.6496	0.3504
R4	0.9938	0.7386	0.2160	0.0453
R5	0.7375	0.0004	0.6380	0.3616
R6	0.0011	0.0487	0.0742	0.8771
R7	0.7026	0.0000	0.8207	0.1793
R8	0.1263	0.0412	0.9588	0.0000
R9	1.0000	0.7382	0.0000	0.2618

**Table 37:** Optimized server for FedProx aggregator rule base: second iteration.

Rule ID	Rule Weight	CN	MCI	AD
R1	0.0000	0.2123	0.7077	0.0800
R2	0.8953	0.0000	0.7188	0.2812
R3	0.4688	0.4743	0.5055	0.0201
R4	0.4004	0.3913	0.2215	0.3872
R5	0.5339	0.0001	0.3351	0.6648
R6	0.4359	0.8704	0.0903	0.0393
R7	0.9764	0.6474	0.0510	0.3016
R8	0.8233	0.0005	0.0981	0.9015
R9	0.0301	0.4195	0.5728	0.0077

**Table 38:** Optimized server for FedProx aggregator rule base: third iteration.

Rule ID	Rule Weight	CN	MCI	AD
R1	0.0489	0.0000	0.1579	0.8421
R2	0.9898	0.7954	0.0000	0.2046
R3	0.2992	0.2640	0.7360	0.0000
R4	0.5001	0.6976	0.0000	0.3024
R5	0.9721	0.4644	0.0000	0.5356
R6	0.5106	0.0000	0.7867	0.2133
R7	1.0000	0.0016	0.5032	0.4952
R8	0.6459	0.7756	0.2244	0.0000
R9	0.7654	0.6747	0.0183	0.3070

**Table 39:** Optimized server for Genetic Algorithm aggregator rule base: first iteration.

Rule ID	Rule Weight	CN	MCI	AD
R1	0.9762	0.0000	0.4092	0.5908
R2	0.4676	0.0000	0.7109	0.2891
R3	0.7043	0.0050	0.9950	0.0000
R4	0.5023	0.1734	0.8266	0.0000
R5	0.0000	0.0453	0.5596	0.3950
R6	0.0000	0.0000	0.3649	0.6351
R7	0.7269	0.0000	0.5659	0.4341
R8	0.9804	0.0434	0.9566	0.0000
R9	0.9975	0.7934	0.2053	0.0013

**Table 40:** Optimized server for Genetic Algorithm aggregator rule base: second iteration.

Rule ID	Rule Weight	CN	MCI	AD
R1	0.2041	0.4060	0.4854	0.1087
R2	0.7601	0.0000	0.8964	0.1036
R3	0.6395	0.7190	0.2281	0.0529
R4	1.0000	0.6089	0.3903	0.0008
R5	0.0390	0.0000	0.0832	0.9168
R6	0.6207	0.0000	0.8847	0.1153
R7	0.3963	0.8816	0.0064	0.1120
R8	0.3521	0.0014	0.2706	0.7280
R9	0.0481	0.2013	0.6028	0.1959

**Table 41:** Optimized server for Genetic Algorithm aggregator rule base: third iteration.

Rule ID	Rule Weight	CN	MCI	AD
R1	0.5367	0.0000	0.4371	0.5629
R2	0.4920	0.0005	0.1798	0.8197
R3	0.2167	0.0175	0.9825	0.0000
R4	0.3923	0.8146	0.0064	0.1790
R5	0.9993	0.0006	0.5347	0.4653
R6	0.0000	0.0001	0.1645	0.8354
R7	0.3796	0.5033	0.0631	0.4336
R8	0.2942	0.1037	0.8963	0.0000
R9	0.0000	0.4387	0.0000	0.5613

**Table 42:** Model performance after last iteration.

Aggregator Client		Training		Testing	
		MSE	Accuracy	MSE	Accuracy
FedAvg Aggregator	Client 1	0.015088	0.40465	0.01072	0.48148
	Client 2	0.02309	0.35814	0.01808	0.37037
	Client 3	0.01404	0.59813	0.01405	0.52830
	Global	0.00409	0.99845	0.00465	0.99999
FedProx Aggregator	Client 1	0.01391	0.59535	0.01040	0.61111
	Client 2	0.03767	0.41395	0.03701	0.38889
	Client 3	0.02491	0.37383	0.02683	0.18868
	Global	0.00592	0.73602	0.00687	0.68944
Genetic Algorithm	Client 1	0.036076	0.31163	0.032451	0.29629
	Client 2	0.04106	0.35348	0.037835	0.24074
	Client 3	0.03331	0.36448	0.03915	0.24528
	Global	0.00833	0.51086	0.00904	0.43478

**Table 43:** PSO time analysis across iterations for FedAvg aggregator.

Iteration	Client 1 Time (s)	Client 2 Time (s)	Client 3 Time (s)	Final PSO Time (s)
1	253.88	375.92	296.97	742.59
2	251.42	249.82	252.57	746.66
3	279.37	252.04	251.23	734.71
4	259.25	251.35	249.78	739.96
5	249.15	249.71	250.31	734.90
Total	1293.07	1129.09	1300.86	3998.82

**Table 44:** Comparison with state-of-the-art methods (X indicates that the issue was not addressed).

Reference	Methodology	Dataset	Accuracy	Privacy-preserving	Uncertainty Issue
Umme et al. [1]	DementiaNet + Transfer Learning	MRI Dataset (6400 Images)	97%	X	X
Raees et al. [40]	SVM + DNN	MCI dataset (111 people)	90%	X	X
Buvaneswari et al. [41]	SegNet + ResNet-101	ADNI dataset	96%	X	X
Saratxaga et al. [42]	ResNet 18 + BrainNet	Collected From Kaggle	80%, 90%	X	X
Hu et al. [43]	CNN	ADNI and NIFD dataset	92%	X	X
Khalil et al. [5]	Hardware Acceleration (VHDL + FPGA)	Simulation Data	89%	Achieved 87% sensitivity; Low power consumption (35–39 mW)	X
Mitrovska et al. [6]	FedAvg, Secure Aggregation (SecAgg)	Demographic Simulations	-	Highlighted privacy guarantees; Evaluated statistical variations	X
Trivedi et al. [32]	Federated Deep Learning + AlexNet	IID Dataset	98.53%	Ensured data privacy; Tested with single/multiple clients	X
Altalbe et al. [33]	DNN within Federated Learning	Kay Elemetrics voice disorder data	82.82%	Reduced overfitting; Preserved privacy and security	X
Mandawkar et al. [34]	Tawny Flamingo Deep CNN	Clinical Data	98.252% (K-fold), 97.995% (training)	High accuracy; Tuned by Tawny Flamingo algorithm	X
Castro et al. [35]	Federated Learning + Biometric Authentication	OASIS, ADNI datasets	Comparable	Protected privacy; Prevented data poisoning	X
Qian et al. [9]	FeDeFo (Deep Forest + Federated Learning)	sMRI images	-	Personalized model while protecting data privacy	X
Proposed Research	Hybrid Deep and Federated Learning	ADNI and NIfTI files dataset	99.9%	Ensured data privacy; Tested with multiple clients	Solved

## Data Availability

The data used to support the findings of this study are available upon reasonable request to the corresponding author.
